# A one-dimensional cadmium(II) complex supported by a sulfur–nitro­gen mixed-donor ligand

**DOI:** 10.1107/S160053680902371X

**Published:** 2009-06-27

**Authors:** Qian Gao, Chao-Yan Zhang, Yue Cui, Ya-Bo Xie

**Affiliations:** a College of Environmental and Energy Engineering, Beijing University of Technology, Beijing 100022, People’s Republic of China

## Abstract

In the title compound, *catena*-poly[cadmium(II)-bis­(μ-5-am­ino-1,3,4-thia­diazole-2-thiol­ato)-κ^2^ *N* ^3^:*S* ^2^;κ^2^ *S* ^2^:*N* ^3^], [Cd(C_2_H_2_N_3_S_2_)_2_]_*n*_, the Cd^II^ ion is coordinated by two N atoms of the 1,3,4-thia­diazole rings from two ligands and two S atoms of sulfhydryl from two other ligands in a slightly distorted tetra­hedral geometry. The ligands bridge Cd^II^ ions, forming one-dimensional chains along [001], which are connected by N—H⋯N and N—H⋯S hydrogen bonds into a three-dimensional network.

## Related literature

For self-assembled coordination polymeric complexes with versatile structure features, see: Mulfort & Hupp(2007 ▶); Liu *et al.* (2003 ▶); Bauer *et al.* (2007 ▶). For the effect of hydrogen bonding in stabilizing and regulating the supra­molecular construction, see: Dalrymple & Shimidzu (2007 ▶); Dong *et al.* (2006 ▶); Wang *et al.* (2005 ▶). For similar stuctures and bond lengths, see: Tzeng, Lee *et al.* (2004 ▶); Tzeng *et al.* (1999 ▶); Tzeng, Huang *et al.* (2004 ▶).
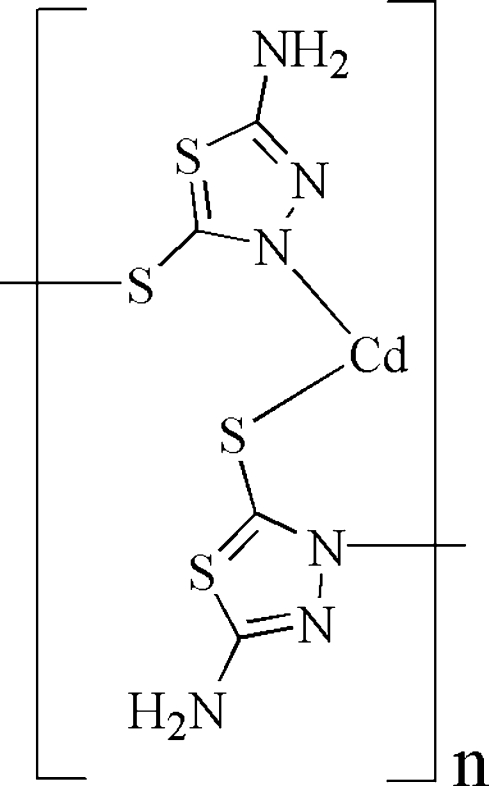


## Experimental

### 

#### Crystal data

[Cd(C_2_H_2_N_3_S_2_)_2_]
*M* *_r_* = 376.77
Monoclinic, 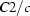

*a* = 12.6419 (11) Å
*b* = 10.8341 (10) Å
*c* = 7.7241 (7) Å
β = 92.795 (1)°
*V* = 1056.66 (16) Å^3^
*Z* = 4
Mo *K*α radiation
μ = 2.83 mm^−1^
*T* = 293 K
0.24 × 0.24 × 0.20 mm

#### Data collection

Bruker SMART CCD area-detector diffractometer
Absorption correction: multi-scan (*SADABS* ; Bruker, 1998 ▶) *T*_min_ = 0.550, *T*_max_ = 0.602 (expected range = 0.519–0.568)
3155 measured reflections
1232 independent reflections
1198 reflections with *I* > 2σ(*I*)
*R*_int_ = 0.015

#### Refinement

*R*[*F* ^2^ > 2σ(*F* ^2^)] = 0.015
*wR*(*F* ^2^) = 0.043
*S* = 1.01
1232 reflections
70 parameters
H-atom parameters constrained
Δρ_max_ = 0.39 e Å^−3^
Δρ_min_ = −0.49 e Å^−3^

### 

Data collection: *SMART* (Bruker, 1998 ▶); cell refinement: *SAINT* (Bruker, 1998 ▶); data reduction: *SAINT*; program(s) used to solve structure: *SHELXS97* (Sheldrick, 2008 ▶); program(s) used to refine structure: *SHELXL97* (Sheldrick, 2008 ▶); molecular graphics: *SHELXTL* (Sheldrick, 2008 ▶); software used to prepare material for publication: *SHELXTL*.

## Supplementary Material

Crystal structure: contains datablocks global, I. DOI: 10.1107/S160053680902371X/pk2170sup1.cif

Structure factors: contains datablocks I. DOI: 10.1107/S160053680902371X/pk2170Isup2.hkl

Additional supplementary materials:  crystallographic information; 3D view; checkCIF report

## Figures and Tables

**Table 1:** Hydrogen-bond geometry (Å, °)

*D*—H⋯*A*	*D*—H	H⋯*A*	*D*⋯*A*	*D*—H⋯*A*
N3—H3*A*⋯N2^i^	0.86	2.25	3.064 (2)	158
N3—H3*B*⋯N2^ii^	0.86	2.66	3.119 (2)	114
N3—H3*B*⋯S1^iii^	0.86	2.74	3.4694 (17)	144

Symmetry codes: (i) 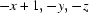; (ii) 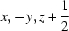; (iii) 
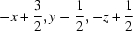
.

**Fig. 1.**
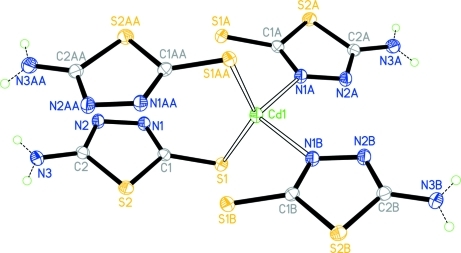
The molecular structure of the title compound with displacement ellipsoids drawn at the 30% probability level for non-hydrogen atoms. Symmetry related atoms have the following symmetry codes: A = x, -y + 1, z + 1/2 B = -x + 1, -y + 1, -z AA= -x + 1, y, -z+1/2.

**Fig. 2.**
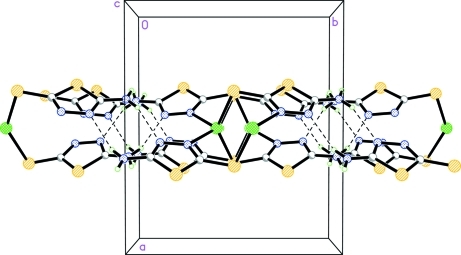
The complexes are linked by N—H···N hydrogen bonds along the c axis.

**Fig. 3.**
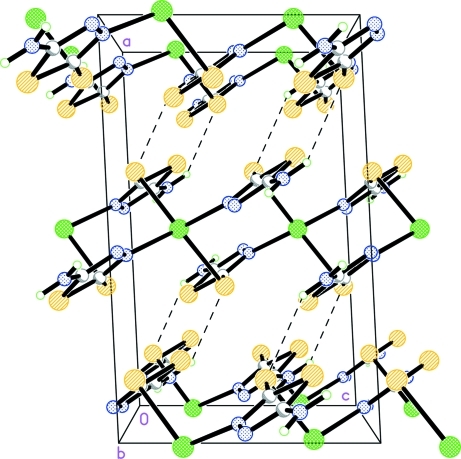
The complexes are connected by N—H···S hydrogen bonds along the a axis.

## supplementary crystallographic information

### Comment

Owing to their potential as new functional materials, interest in self-assembled coordination polymeric complexes with versatile structure features has grown rapidly (Mulfort *et al.*, 2007; Liu *et al.*, 2003; Bauer *et al.*, 2007). Hydrogen bonding is one highly directional supramolecular force, and although weaker than coordinative bonds, have been recognized to play critical roles in stabilizing and regulating the supramolecular construction (Dalrymple *et al.*, 2007). Crystal engineering studies of hydrogen bonding in low-dimensional materials, especially in one-dimensional transition metal complexes, have been reported by several groups (Dong *et al.*, 2006; Wang *et al.*, 2005). Tzeng and coworkers have reported 2-amino-5-mercapto-1,3,4-thiadiazolate (*L*), acting as an auxiliary ligand and displaying its active coordination properties with Pd(II) (Tzeng, Lee *et al.*, 2004) and Au(I) (Tzeng *et al.*, 1999; Tzeng, Huang *et al.*, 2004) to form diverse crystal structures. The various hydrogen bonding interactions have also been investigated, and have shown important effects in forming large molecular arrays. However, in these compounds, the ligand had unidentate coordination to metal ions with the sulfur atom of sulfhydryl. Herein, we report the crystal structure of Cd^II^ complex, [Cd(C_2_H_2_N_3_S_2_)_2_]_n_ (I), using 2-amino-5-mercapto-1,3,4-thiadiazolate (*L*) as the unique bridging ligand and exhibiting one-dimensional chain structure feature.

A perspective view of a tetranuclear fragment of the chain is shown in Fig. 1. There is one crystallographically independent Cd^II^ ion coordinated to two nitrogen atoms which belong to the 1,3,4-thiadiazole rings from two ligands, with N1A—Cd1—N1B angle of 103.50 (7)°, two sulfur atoms of sulfhydryl from two other ligands with S1—Cd1—S1A angle of 139.05 (2)°, and displaying a slightly distorted tetrahedron geometry. The bond length of Cd—S is 2.5264 (4) Å, which is significantly longer than that of unidentate coordination to metal ions (Pd—S 2.2793 (9) Å, Tzeng, Lee *et al.*, 2004) (Au—S 2.295 (5)–2.323 (4) Å, Tzeng *et al.*, 1999; Tzeng, Huang *et al.*, 2004). Nitrogen atoms participating in coordination may cause the Cd—S bond to lengthen. Simultaneously, each ligand bridges two Cd^II^ ions to from a one-dimensional chain along the *c* axis.

There are two kinds of hydrogen bond in the complex. N—H···N hydrogen bonds exist between the hydrogen atom of the amidogen from one chain and the uncoordinated nitrogen atom of the 1,3,4-thiadiazole ring from the adjacent chain. This joins the chains along the *c* axis into a two-dimensional plane (Fig. 2).  N—H···S hydrogen bonds occur between the other hydrogen atom of the same amidogen and the sulfur atom of the coordinated sulfhydryl from an adjacent chain. This joins the one-dimensional chains along the *a* axis to create a two-dimensional plane  (Fig. 3). The parameters of hydrogen bonds are given in the Table 1.

### Experimental

A mixture of 2-amino-5-mercapto-1,3,4-thiadiazole (39.95 mg, 0.3 mmol) (HL), LiOH.H_2_O (12.59 mg, 0.3 mmol) and Cd(NO_3_)_2_.4H_2_O (92.55 mg, 0.3 mmol) was dissolved in 25 ml MeOH/H_2_O. The resulting solution was filtered and the filtrate was allowed to stand for several days. Light yellow crystals were collected in about 30% yield (based on Cd^II^).

### Refinement

H atoms of N were located in Fourier difference maps and refined with isotropic displacement parameters set at 1.2 times those of the parent N atoms.

### Crystal data

**Table:**

[Cd(C_2_H_2_N_3_S_2_)_2_]	*F*(000) = 728
*M**_r_* = 376.77	*D*_x_ = 2.368 Mg m^−^^3^
Monoclinic, *C*2/*c*	Mo *K*α radiation, λ = 0.71073 Å
Hall symbol: -C 2yc	Cell parameters from 2746 reflections
*a* = 12.6419 (11) Å	θ = 2.5–27.9°
*b* = 10.8341 (10) Å	µ = 2.83 mm^−^^1^
*c* = 7.7241 (7) Å	*T* = 293 K
β = 92.795 (1)°	Block, colorless
*V* = 1056.66 (16) Å^3^	0.24 × 0.24 × 0.20 mm
*Z* = 4	

### Data collection

**Table:**

Bruker SMART CCD area-detector diffractometer	1232 independent reflections
Radiation source: fine-focus sealed tube	1198 reflections with *I* > 2σ(*I*)
graphite	*R*_int_ = 0.015
φ and ω scans	θ_max_ = 27.9°, θ_min_ = 2.5°
Absorption correction: multi-scan (*SADABS* ; Bruker, 1998)	*h* = −11→16
*T*_min_ = 0.550, *T*_max_ = 0.602	*k* = −14→14
3155 measured reflections	*l* = −10→10

### Refinement

**Table:**

Refinement on *F*^2^	Secondary atom site location: difference Fourier map
Least-squares matrix: full	Hydrogen site location: inferred from neighbouring sites
*R*[*F*^2^ > 2σ(*F*^2^)] = 0.015	H-atom parameters constrained
*wR*(*F*^2^) = 0.043	*w* = 1/[σ^2^(*F*_o_^2^) + (0.0274*P*)^2^ + 0.7843*P*] where *P* = (*F*_o_^2^ + 2*F*_c_^2^)/3
*S* = 1.01	(Δ/σ)_max_ = 0.001
1232 reflections	Δρ_max_ = 0.39 e Å^−^^3^
70 parameters	Δρ_min_ = −0.49 e Å^−^^3^
0 restraints	Extinction correction: *SHELXL97* (Sheldrick, 2008), Fc^*^=kFc[1+0.001xFc^2^λ^3^/sin(2θ)]^-1/4^
Primary atom site location: structure-invariant direct methods	Extinction coefficient: 0.0116 (5)

### Special details

**Table:**

Geometry. All e.s.d.'s (except the e.s.d. in the dihedral angle between two l.s. planes) are estimated using the full covariance matrix. The cell e.s.d.'s are taken into account individually in the estimation of e.s.d.'s in distances, angles and torsion angles; correlations between e.s.d.'s in cell parameters are only used when they are defined by crystal symmetry. An approximate (isotropic) treatment of cell e.s.d.'s is used for estimating e.s.d.'s involving l.s. planes.
Refinement. Refinement of *F*^2^ against ALL reflections. The weighted *R*-factor *wR* and goodness of fit *S* are based on *F*^2^, conventional *R*-factors *R* are based on *F*, with *F* set to zero for negative *F*^2^. The threshold expression of *F*^2^ > 2σ(*F*^2^) is used only for calculating *R*-factors(gt) *etc*. and is not relevant to the choice of reflections for refinement. *R*-factors based on *F*^2^ are statistically about twice as large as those based on *F*, and *R*- factors based on ALL data will be even larger.

### Fractional atomic coordinates and isotropic or equivalent isotropic displacement parameters (Å^2^)

**Table:**

	*x*	*y*	*z*	*U*_iso_*/*U*_eq_	
Cd1	0.5000	0.582614 (14)	0.2500	0.02585 (9)	
C1	0.62301 (13)	0.34405 (15)	0.0808 (2)	0.0238 (3)	
C2	0.61171 (13)	0.12413 (16)	0.1243 (2)	0.0267 (3)	
N1	0.56499 (11)	0.28638 (13)	−0.03734 (18)	0.0262 (3)	
N2	0.55650 (12)	0.16022 (13)	−0.01418 (19)	0.0289 (3)	
N3	0.61723 (13)	0.00640 (15)	0.1767 (2)	0.0384 (4)	
H3A	0.5834	−0.0499	0.1182	0.046*	
H3B	0.6546	−0.0129	0.2687	0.046*	
S1	0.64750 (3)	0.50104 (4)	0.07269 (5)	0.02700 (11)	
S2	0.67601 (4)	0.24382 (4)	0.23801 (6)	0.03215 (12)	

### Atomic displacement parameters (Å^2^)

**Table:**

	*U*^11^	*U*^22^	*U*^33^	*U*^12^	*U*^13^	*U*^23^
Cd1	0.03358 (12)	0.02365 (12)	0.02017 (11)	0.000	−0.00030 (7)	0.000
C1	0.0249 (7)	0.0262 (8)	0.0202 (7)	0.0030 (6)	−0.0006 (5)	0.0004 (6)
C2	0.0242 (7)	0.0278 (8)	0.0282 (8)	0.0013 (6)	0.0016 (6)	0.0024 (6)
N1	0.0326 (7)	0.0236 (7)	0.0219 (6)	−0.0002 (5)	−0.0033 (5)	−0.0001 (5)
N2	0.0349 (7)	0.0234 (7)	0.0281 (7)	0.0002 (6)	−0.0027 (6)	0.0004 (5)
N3	0.0346 (8)	0.0297 (8)	0.0500 (10)	−0.0012 (6)	−0.0079 (7)	0.0144 (7)
S1	0.0292 (2)	0.0257 (2)	0.0261 (2)	−0.00241 (15)	0.00115 (15)	−0.00137 (15)
S2	0.0341 (2)	0.0325 (2)	0.0287 (2)	−0.00058 (17)	−0.01070 (17)	0.00401 (16)

### Geometric parameters (Å, °)

**Table:**

Cd1—N1^i^	2.2927 (14)	C2—N2	1.308 (2)
Cd1—N1^ii^	2.2927 (14)	C2—N3	1.339 (2)
Cd1—S1	2.5264 (4)	C2—S2	1.7446 (18)
Cd1—S1^iii^	2.5264 (4)	N1—N2	1.383 (2)
C1—N1	1.302 (2)	N1—Cd1^ii^	2.2927 (14)
C1—S1	1.7304 (17)	N3—H3A	0.8600
C1—S2	1.7390 (16)	N3—H3B	0.8600
			
N1^i^—Cd1—N1^ii^	103.50 (7)	N3—C2—S2	122.69 (13)
N1^i^—Cd1—S1	110.90 (4)	C1—N1—N2	115.35 (13)
N1^ii^—Cd1—S1	94.38 (4)	C1—N1—Cd1^ii^	112.01 (11)
N1^i^—Cd1—S1^iii^	94.38 (4)	N2—N1—Cd1^ii^	132.61 (10)
N1^ii^—Cd1—S1^iii^	110.90 (4)	C2—N2—N1	111.06 (15)
S1—Cd1—S1^iii^	139.05 (2)	C2—N3—H3A	120.0
N1—C1—S1	122.83 (12)	C2—N3—H3B	120.0
N1—C1—S2	111.98 (12)	H3A—N3—H3B	120.0
S1—C1—S2	125.13 (9)	C1—S1—Cd1	100.73 (6)
N2—C2—N3	123.33 (17)	C1—S2—C2	87.62 (8)
N2—C2—S2	113.98 (13)		
			
S1—C1—N1—N2	177.84 (12)	S2—C1—S1—Cd1	−90.15 (11)
S2—C1—N1—N2	0.51 (19)	N1^i^—Cd1—S1—C1	128.77 (6)
S1—C1—N1—Cd1^ii^	−0.59 (17)	N1^ii^—Cd1—S1—C1	−124.98 (6)
S2—C1—N1—Cd1^ii^	−177.93 (7)	S1^iii^—Cd1—S1—C1	4.38 (5)
N3—C2—N2—N1	−179.98 (16)	N1—C1—S2—C2	0.11 (13)
S2—C2—N2—N1	1.14 (19)	S1—C1—S2—C2	−177.15 (12)
C1—N1—N2—C2	−1.1 (2)	N2—C2—S2—C1	−0.74 (14)
Cd1^ii^—N1—N2—C2	176.95 (12)	N3—C2—S2—C1	−179.63 (16)
N1—C1—S1—Cd1	92.87 (14)		

Symmetry codes: (i) *x*, −*y*+1, *z*+1/2; (ii) −*x*+1, −*y*+1, −*z*; (iii) −*x*+1, *y*, −*z*+1/2.

### Hydrogen-bond geometry (Å, °)

**Table:**

*D*—H···*A*	*D*—H	H···*A*	*D*···*A*	*D*—H···*A*
N3—H3A···N2^iv^	0.86	2.25	3.064 (2)	158
N3—H3B···N2^v^	0.86	2.66	3.119 (2)	114
N3—H3B···S1^vi^	0.86	2.74	3.4694 (17)	144

Symmetry codes: (iv) −*x*+1, −*y*, −*z*; (v) *x*, −*y*, *z*+1/2; (vi) −*x*+3/2, *y*−1/2, −*z*+1/2.
